# Clinical characteristics and treatment outcomes in a cohort of patients with pyogenic and amoebic liver abscess

**DOI:** 10.1186/s12879-019-4127-8

**Published:** 2019-06-03

**Authors:** Lorna Neill, Frances Edwards, Simon M. Collin, David Harrington, Dominic Wakerley, Guduru Gopal Rao, Alastair C. McGregor

**Affiliations:** 1 Department of Infectious Diseases and Tropical Medicine, London North West Healthcare NHS Trust, Northwick Park Hospital, Middlesex, Harrow, HA1 3UJ UK; 2 Healthcare-Associated Infection & Antimicrobial Resistance Division, National Infection Service, Public Health England, 61 Colindale Avenue, London, NW9 5EQ UK; 3 Department of Microbiology, London North West Healthcare NHS Trust, Northwick Park Hospital, Middlesex, Harrow, HA1 3UJ UK; 4 Department of Medicine, Imperial College London, London, UK

**Keywords:** Liver abscess, Amoebic liver abscess, Pyogenic liver abscess, 16S ribosomal RNA, Risk factors, Treatment outcome, Hospital mortality

## Abstract

**Background:**

We describe the clinical features of a cohort of patients with liver abscesses and investigate relationships between clinical, radiological and microbiological findings and mortality.

**Methods:**

Retrospective review of pyogenic (PLA) or amoebic liver abscesses (ALA) diagnosed and treated at a major infectious diseases department in London over 9 years.

**Results:**

One hundred forty-one patient records were identified; 132 (93.6%) had PLA and 9 (6.4%) ALA. No organism was identified in 38.6% (51/132); a single bacterial species was isolated in 47.0% (62/132) of PLA, ≥ 2 in 14.4% (19/132). There was weak evidence of variation in abscess size by type of microorganism, with streptococcal PLA typically larger (*p* = 0.03 for *Streptococcus milleri* group, *p* = 0.05 for non-milleri streptococci). Patients with ALA were younger (median 41, IQR 37–51 years) than those with PLA (median 68, IQR 50.5–78 years) (*p* = 0.003) and all were male (9/9, 100%, (*p* = 0.03)), with a history of recent travel in the majority (6/9, 66.7% (*p* = 0.003)). C-reactive protein was higher in ALA than in PLA (*p* = 0.06). In the entire cohort, loculation (HR = 2.51 (95% CI 1.00–6.32), *p* = 0.04) and baseline ALP (HR = 4.78 (95% CI 1.19–19.2) per log_10_ increase, *p* = 0.03) were associated with mortality. 16S ribosomal RNA (rRNA) analysis was used in a subset of culture-negative cases and increased the diagnostic yield by 13%.

**Conclusions:**

Clinical or radiological features cannot be used to distinguish between PLA and ALA, or help identify the bacterial cause of PLA. However, ALA is more common in young, male patients with a history of travel. 16S rRNA analysis of abscess fluid has a role in improving microbiological diagnosis in culture-negative cases.

**Electronic supplementary material:**

The online version of this article (10.1186/s12879-019-4127-8) contains supplementary material, which is available to authorized users.

## Background

The incidence of liver abscess, a rare but potentially life-threatening infection, appears to be increasing worldwide [1–4]. The use of antibiotics, imaging and less invasive procedures for source control have improved treatment outcomes over the last century, but mortality remains high [1, 5, 6] At present, there are no national or international clinical guidelines, and limited evidence to guide decisions about care.

A wide variety of bacteria have been described as causes of pyogenic liver abscess (PLA), but published data on associations of bacterial species with clinical presentation, radiological findings and prognosis are sparse. *Entamoeba histolytica*, a protozoan parasite, is also a well-recognized non-bacterial cause of liver abscess. In Europe, cases of amoebic liver abscess (ALA) are rare and usually imported, whereas in some highly endemic areas ALA can be more common than PLA [7]. There is good evidence that ALA can be treated successfully with shorter courses of antimicrobial therapy than PLA, and that drainage is generally not required [8]. However, there is limited evidence as to how PLA and ALA can be distinguished clinically or radiologically.

In this study we describe a cohort of liver abscess patients treated at a large infectious diseases department over a 9-year period and report the clinical characteristics and treatment outcomes of PLA and ALA.

## Methods

### Setting and patients

We conducted a retrospective case note review of all adult liver abscess patients treated at our hospital (Northwick Park Hospital, Middlesex, UK) between May 2008 and June 2017. Patients presenting with liver abscess were identified using a prospectively recorded database of infectious disease inpatients, as well as a retrospective search of clinical coding data (search term “liver abscess”, ICD 10 code = K750). Data collected from the hospital electronic patient information systems included patient characteristics (age, sex, ethnicity), medical background and comorbidities, the results of standard biochemical and haematological tests on admission (full blood count, C-Reactive Protein (CRP), alanine transaminase (ALT), alkaline phosphatase (ALP) and bilirubin), the characteristics of the abscess (isolated microorganism(s) and resistance patterns, number of abscesses, maximum diameter and loculation), and treatment outcome (antibiotic use, use of aspiration, discharge, death).

### Management of liver abscess

In the absence of consensus guidelines, patients with liver abscesses were investigated and managed at the discretion of the attending physician. Abscesses were identified by ultrasound or computed tomography. Samples submitted to the microbiology lab (abscess pus, blood cultures) were processed according to national guidelines (British Society of Antimicrobial Chemotherapy (BSAC), until February 2016, then European Committee on Antimicrobial Susceptibility Testing (EUCAST)) in a CPA (Clinical Pathology Accreditation) accredited laboratory. The choice of antimicrobial agents was guided by infection specialists. In a subset of cases, aspirates were sent for 16S ribosomal RNA (rRNA) sequencing (Great Ormond Street Hospital, in-house assay). Amoebic serology was performed when there was clinical suspicion of *E. histolytica* infection (in-house immunofluorescent antibody test (IFAT) and Cellulose Acetate Precipitin (CAP), Department of Parasitology, Hospital for Tropical Diseases, London).

### Statistical analysis

Characteristics of pyogenic and amoebic abscesses were compared using Fisher’s exact and Kruskal-Wallis tests for categorical and continuous variables, respectively (*α* = 0.05). Differences in clinical characteristics and treatment outcome (death within 30 days, and death within 6 months) by type of isolated microorganism were tested using a Wald-type test (F statistic) of Somers’ D parameters for continuous variables (adjusted for within-patient clustering) or Fisher’s exact tests for categorical variables. Associations of type of microorganism with all-cause mortality (within 30 days and within 6 months of admission) were investigated by fitting random-effects parametric survival-time models with patient as the panel variable and standard errors adjusted for within-patient clustering. Risk factors for 6-month all-cause mortality were investigated using Cox regression models. Variables for which there was evidence (*p* ≤ 0.1) suggestive of an association in univariate models were carried forward to a multivariable model, from which variables were eliminated by mutual adjustment to obtain a final model of independently associated (*p* ≤ 0.05) risk factors.

## Results

### Characteristics of PLA and ALA patients

We identified 132 pyogenic liver abscess (PLA) and 9 amoebic liver abscess (ALA) admissions during the period May 2008 to June 2017 (Table 1). PLA patients were representative of the ethnically diverse local population: 47/132 (35.6%) identified as “White (British/other)”; 33/132 (25%) as “Indian/Pakistani”; and 19/132 (14.4%) as “Other Asian”. All but one of the ALA patients was from an “Indian/Pakistani” (5/9, 55.6%) or “Other Asian” background (3/9, 33.3%)**.**

**Table 1:** Characteristics of patients (*N* = 141) by type of liver abscess (pyogenic or amoebic)

		Pyogenic (*n* = 132)	Amoebic (*n* = 9)
Sex	Male	84 (63.6%)	9 (100.0%)
Age (years)	Median (IQR)	68 (51–78)	41 (37–51)
Ethnicity	White (British or Other)	47 (35.6%)	1 (11.1%)
	Indian/Pakistani	33 (25.0%)	5 (55.6%)
	Asian	19 (14.4%)	3 (33.3%)
	Other	17 (12.9%)	0 (0.0%)
	Caribbean	13 (9.9%)	0 (0.0%)
	African	3 (2.3%)	1 (11.1%)
Number of organisms^a^	0	51 (38.6%)	–
	1	62 (47.0%)	–
	2	9 (6.8%)	–
	3	8 (6.1%)	–
	4	2 (1.5%)	
Organism	None	51 (38.6%)	–
Grouped for analysis	Enterobacteriaceae	14 (10.6%)	–
	*E. Coli*	21 (15.9%)	
	*Klebsiella* spp.	21 (15.9%)	
	*S. milleri* group	19 (14.4%)	–
	Other *Strep* spp.	7 (5.3%)	
	Anaerobe	16 (12.1%)	–
Grouped as ‘other’ for analysis	*Enterococcus*	5 (3.8%)	
	*Staphylococcus aureus*	1 (0.8%)	–
	*Pseudomonas aeriginosa*	2 (1.5%)	–
	Other (*Anaeroglobus*, *Haemophilus, Corynebacterium*, *Sutterella*)	5 (3.8%)	–
Number of abscesses identified	1	75 (56.8%)	7 (77.8%)
	2	19 (14.4%)	1 (11.1%)
	≥3	38 (28.8%)	1 (11.1%)
Maximum diameter (cm)	Median (IQR)	6.1 (4.8–9.0), *n* = 118	7.3 (5.8–8.8), *n* = 8
Loculated		52 (39.4%)	1 (11.1%)
Antimicrobial resistance^b^		45 (34.1%)	–
Abscess aspirated/drained		79 (59.9%)	6 (66.7%)
Diabetic		36 (27.3%)	3 (33.3%)
Recent travel (< 1 year)		23 (21.1%)	6 (66.7%)
Immunosuppression (chemotherapy, steroids, immunotherapy or combination in previous year)		18 (13.6%)	0 (0.0%)
Died within 30 days		7 (5.3%)	0 (0.0%)
Died within 6 months		20 (15.2%)	0 (0.0%)

^a^in total, 111 microorganisms were isolated from 81 pyogenic liver abscess patients

^b^Resistance was detected to one or more antimicrobial agents in 45/132 patients and in 54/111 (48.7%) of microorganisms

ALA patients were younger (median 41, IQR 37–51 years) than PLA patients (median 68, IQR 50.5–78 years) (*p* = 0.003), and all ALA patients were male compared with 63.6% (84/132) of PLA patients (*p* = 0.03). 66.7% (6/9) of ALA patients had documentation of recent travel (< 1 year) compared to only 21.1% (23/132) of PLA patients (*p* = 0.003)**.** Coexisting diabetes mellitus was recorded for 27.3% (36/132) of PLA and 33.3% (3/9) of ALA patients. Eighteen PLA patients (13.6%) and none of the ALA patients were classed as immunocompromised in the preceding year. None of the patients were known to be HIV positive.

### Radiological characteristics of PLA and ALA

There were no differences in the radiological characteristics of ALA compared with PLA: multiple abscesses, ALA 22.2% (2/9), PLA 43.2% (57/132), *p* = 0.30; maximum diameter, ALA median 7.3 cm (IQR 5.8–8.8 cm), PLA 6.1 cm (4.8–9.0 cm), *p* = 0.50; loculation, ALA 11.1% (1/9), PLA 39.4% (52/132), *p* = 0.15, or position, ALA right sided abscess 77.8% (7/9), PLA 68.6% (81/118), *p* = 0.7 (Additional file 1: Table S1).

### PLA microbiology

Organisms were identified via culture from peripheral blood (33/132, 25%) or from pus aspirated from the abscesses (45/132, 34%) or from both. 16S rRNA gene sequencing was available from early 2015, and was performed on 17 abscesses, giving a positive result in 14 cases; in 9/14 cases it was the only method by which an organism was identified (Additional file 1: Table S2). A single bacterial species was isolated in 47.0% (62/132) of PLA. Multiple pathogens were identified in 19/132 (14.4%). In 51/132 (38.6%), no microbiological cause was found. The most common bacteria identified were: *Escherichia coli* (15.9%, 21/132); *Klebsiella* spp. (15.9%, 21/132); and *Streptococcus milleri* group (14.4%, 19/132) (Table 1).

### Clinical characteristics by type of microorganism

There was weak evidence for overall variation in size (*p* = 0.04) and loculation (*p* = 0.09) of abscesses by type of microorganism (Table 2). Abscesses caused by streptococci (*S. milleri* group and non-milleri streptococci) tended to be larger than those caused by other microorganisms (Somers’ D *p* = 0.03 for *S. milleri*, *p* = 0.05 for non-milleri streptococci). Abscesses with no isolated microorganism tended to be smaller (*p* = 0.04). There were no overall associations between specific microorganisms and baseline biochemical measurements (Table 2), but compared with all other microorganism: Enterobacteriaceae (including *E. coli*) and *S. milleri* were associated with lower ALT (Somers’ D *p* = 0.03 and *p* = 0.05, respectively), and *Klebsiella* with higher ALT (*p* < 0.001). ALA tended to be associated with higher CRP (*p* = 0.06).

**Table 2:** Clinical characteristics by type of microorganism^a^

		None (*n* = 51)	Enterobacteriaceae including *E. coli* (*n* = 35)	*Klebsiella* (*n* = 21)	*S. milleri* (*n* = 19)	Other *Strep* spp. (*n* = 7)	Anaerobe (*n* = 16)	Other (*n* = 13)	Amoebic (*n* = 9)	*p*-value^b^
Abscess										
Number of abscesses	1	27 (52.9%)	19 (54.3%)	13 (61.9%)	12 (63.2%)	5 (71.4%)	8 (50.0%)	6 (46.2%)	7 (77.8%)	*p* = 0.80
	2+	24 (47.1%)	16 (45.7%)	8 (38.1%)	7 (36.8%)	2 (28.6%)	8 (50.0%)	7 (53.9%)	2 (22.2%)	
Max diameter	(cm)	5.4 (3.9, 7.5)	6.0 (4.3, 8.0)	8.0 (5.3, 9.0)	8.5 (5.7, 12.0)	8.0 (6.8, 10.0)	6.0 (3.3, 7.5)	7.3 (5.6, 8.3)	7.3 (5.8, 8.8)	*p* = 0.04
Loculated	Yes	15 (29.4%)	15 (42.9%)	11 (52.4%)	10 (52.6%)	4 (57.1%)	3 (18.8%)	7 (53.9%)	1 (11.1%)	*p* = 0.09
Resistance^c^	Yes	–	26 (74.3%)	15 (71.4%)	3 (15.8%)	1 (14.3%)	2 (12.5%)	7 (53.9%)	–	*p* < 0.001
Other pathology^d^										
None		32 (62.8%)	20 (57.1%)	17 (81.0%)	13 (68.4%)	5 (71.4%)	10 (62.5%)	7 (53.9%)	9 (100.0%)	*p* = 0.52
Biliary malignancy		4 (7.8%)	2 (5.7%)	0 (0.0%)	0 (0.0%)	0 (0.0%)	1 (6.3%)	0 (0.0%)	0 (0.0%)	
Non-malignant biliary		4 (7.8%)	6 (17.1%)	1 (4.8%)	2 (10.5%)	0 (0.0%)	0 (0.0%)	0 (0.0%)	0 (0.0%)	
Non-malignant GI		10 (19.6%)	6 (17.1%)	2 (9.5%)	4 (21.1%)	2 (28.6%)	4 (25.0%)	6 (46.2%)	0 (0.0%)	
Other cancer		1 (2.0%)	1 (2.9%)	1 (4.8%)	0 (0.0%)	0 (0.0%)	1 (6.3%)	0 (0.0%)	0 (0.0%)	
Biochemistry on admission										
WBC (× 10^9^/L)		14.8 (10.8, 17.1)	16.6 (12.2, 21.7)	14.8 (12.9, 16.9)	16.0 (12.9, 20.0)	16.4 (10.6, 21.3)	14.6 (10.6, 20.3)	14.5 (10.5, 19.3)	16.5 (13.9, 19.2)	*p* = 0.61
Neutrophils (×10^9^/L)		11.9 (8.0, 13.9)	14.5 (8.9, 19.3)	13.0 (9.9, 14.3)	13.8 (11.3, 19.3)	13.1 (8.4, 13.5)	12.9 (8.5, 18.8)	12.3 (8.9, 17.5)	12.9 (9.3, 14.6)	*p* = 0.52
Hb (g/L)		117 (102, 134)	109 (96, 128)	110 (106, 121)	113 (101, 131)	105 (100, 120)	107 (100, 129)	105 (99, 141)	128 (118, 130)	*p* = 0.62
CRP (mg/L)		215 (106, 279)	204 (69, 286)	228 (150, 276)	223 (119, 286)	245 (138, 333)	188 (90, 258)	173 (78, 263)	260 (207, 408)	*p* = 0.40
INR		1.20 (1.04, 1.44)	1.30 (1.10, 1.40)	1.20 (1.17, 1.45)	1.22 (1.10, 1.48)	1.30 (1.12, 1.40)	1.12 (1.00, 1.40)	1.12 (1.10, 1.45)	1.17 (1.07, 1.30)	*p* = 0.59
ALT (IU/L)		44 (20, 104)	35 (15, 69)	78 (48, 143)	30 (24, 46)	47 (20, 132)	43 (22, 115)	76 (48, 141)	40 (25, 65)	*p* = 0.003
ALP (IU/L)		146 (91, 252)	170 (98, 268)	186 (118, 297)	168 (111, 250)	237 (62, 319)	158 (105, 228)	297 (105, 319)	156 (125, 225)	*p* = 0.79
BILI (umol/L)		15 (10, 24)	20 (11, 35)	20 (11, 45)	16 (9, 29)	9 (9, 61)	14 (11, 44)	34 (10, 77)	16 (11, 17)	*p* = 0.66

^a^Of the 141 patients, 51 patients had no microorganism isolated, 81 had 1 or more bacterial spp. (120 in total) and 9 patients had amoebic liver abscesses

^b^For continuous measures, F statistic comparing Somer’s D parameters for association of measure with organism type, adjusted for clustering within patients; for categorical variables, Fisher’s exact test

^c^Resistance detected to one or more antimicrobial agents; overall, resistance was detected in 54/111 (48.7%) of isolated microorganisms

^d^Biliary Malignancies (cholangiocarcinoma); other biliary pathology (gallstones, cholangitis/cholecystitis); Non-malignant GI pathology (diverticulitis, fistula, portal vein thrombosis); Other cancer (metastatic melanoma, colorectal cancer, pancreatic cancer)

### Treatment

73.7% (87/118) of PLA had a diameter ≥ 5 cm, compared with 87.5% (7/8) of ALA (*p* = 0.68). Overall, 76.3% (71/93) of abscesses ≥5 cm were drained, compared with 25.0% (8/32) of abscesses < 5 cm. There were 26 abscesses ≥10 cm in diameter, including 1 amoebic abscess. In total, 78 (59.1%) of PLA had aspiration/therapeutic drainage, and 9 (6.9%) required a repeat aspiration/drainage. Seven PLA patients (5.3%) required open surgical drainage; 6/9 (66.7%) ALA patients had an aspiration/drainage of their abscess, but none required further intervention. Length of stay was much shorter for ALA (median 7, IQR 6–9 days) than PLA patients (median 17, IQR 10–29 days) (*p* = 0.004). Antibiotic treatment in the PLA group was continued for a median of 53 days and needed changing at least once in 79.5% (105/132) of patients, two or more times in 34.1% (45/132). Antimicrobial resistance was detected in 34.1% (45/132) of patients, and in 48.7% (54/111) of isolated microorganisms, almost exclusively in enterobacteriaceae (74.3% (26/35)) and *Klebsiella* (71.4% (15/21)). Resistance in the PLA group to the most commonly used first line empiric antibiotics; co-amoxiclav, piperacillin/tazobactam and metronidazole was 12.1% (16/132), 1.5% (2/132) and 0% respectively **(**Table 2**)**.

### Associations of type of microorganism and other factors with death

There were no deaths in the ALA group, compared to 5.3% (7/132) 30-day mortality and 15.2% (20/132) 6-month mortality in the PLA group. Two of the seven 30-day deaths and 4/20 6-month deaths were patients with biliary malignancy. There were no associations between type of microorganism and all-cause mortality within 30 days or 6 months (Additional file 1: Table S3). In univariate analyses, only age was associated with death < 6 months (hazard ratio (HR) = 1.05 (95% CI 1.02, 1.09) per year increase, *p* = 0.003). Loculation, baseline anaemia (Hb < 110 g/L), baseline ALP, and immunosuppression (chemotherapy, steroids, immunotherapy) met the criteria (*p* ≤ 0.1) for evaluation by multivariable analysis. Loculation (HR = 2.51 (95% CI 1.00, 6.32), *p* = 0.04) and baseline ALP (HR = 4.78 (95% CI 1.19, 19.2) per log_10_, i.e. tenfold, increase, *p* = 0.03) (Additional file 1: Table S4) were independently associated with mortality when mutually adjusted.

## Discussion

In our cohort of patients with liver abscess we found no large differences in clinical parameters between pyogenic liver abscess (PLA) and amoebic liver abscess (ALA), other than weak evidence of higher baseline C-Reactive Protein (CRP) for ALA compared with PLA. PLA patients had a 6-month mortality of 15.2% (20/132), whereas no ALA patients died. We found that streptococci were associated with larger abscesses than other bacteria. Baseline ALP and loculation, but not type of microorganism, were associated with increased risk of 6-month all-cause mortality in the PLA group.

Previous studies comparing pyogenic and amoebic abscesses have attempted to identify features that distinguish the two, other than travel history [7, 9–12]. Many reports have shown a preponderance of ALA in male patients, who also tend to be younger than patients with PLA [4, 6, 9–18]. Others have documented differences in shape, position and echogenicity on ultrasonography that suggest ALA over PLA [10, 12, 19, 20]. Our results are consistent with the epidemiology described in these earlier papers, as all of our ALA patients were male and had a median age 30 years younger than the PLA patient group. As in other studies, all 9 ALAs in our cohort involved the right lobe (2 also involved the left). We also found that CRP was higher in ALA than in PLA, although this result is unlikely to assist in differentiating these entities.

Half (47%) of PLAs in our study were monomicrobial, and the most common bacterial isolates (found in roughly equal proportions) were *E. coli, S. milleri* group and *Klebsiella* species, consistent with results from a number of other studies [1, 16, 17, 21, 22]. Culture of fluid obtained by cyst aspiration provided a microbiological diagnosis in 34% of cases, whereas blood culture was only positive in 25%. Given the importance of Enterobacteriaceae as etiologic agents in PLA, and the associated problems of antibiotic resistance, aspiration for diagnostic purposes would appear to be a crucial tool in optimising management. There is also likely to be a role for routine testing of culture-negative aspirates with newer molecular techniques such as PCR amplification and sequencing of the 16S ribosomal RNA (rRNA) gene. This has been shown to be more sensitive than traditional culture methods [23, 24], particularly when antibiotics have been administered [25]. In our cohort, 16S rRNA PCR was the only method resulting in a microbiological diagnosis in 9/14 (64.2%) cases in which it was used. Overall, this technique increased pathogen detection by 13% and only 3/17 (17.6%) samples were negative. Although 16S rRNA analysis does not provide antibiotic susceptibility data, the risk of resistance can be inferred from the identity of the pathogen. Associations between radiological features and type of organism were weak, although we did find that abscesses caused by streptococci tended to be larger than abscesses caused by other bacterial species.

Biliary abnormalities are estimated to be responsible for 30–50% of cases of PLA and are more common than those related to portal pyaemia, or haematogenous or direct spread from an adjacent viscus [5, 12, 26–28]. In our cohort, a biliary source was demonstrated in only 18/132 (13.6%). The reason biliary sources were less common in our cohort than in other series is not clear. Diabetes appears to be a major risk factor for developing PLA, and is associated with severe disease [29–31]. About 30% of our patients with both ALA and PLA were diabetic and it is possible that cases in this group are over-represented in our cohort. The source of infection remains unclear in significant number of PLAs [1, 15, 16] and we were unable to demonstrate a source of infection in almost 40%. A source of particular concern is GI malignancy, as these can easily be missed on initial imaging and reports have documented occult colonic malignancies in approximately 24% of cryptogenic PLAs, suggesting that luminal imaging or colonoscopy should be seriously considered in individuals with PLA without an obvious source [32].

Mortality rates from PLA have declined over time [5, 33]. Recent retrospective studies report mortalities between 0 and 13%, although length of follow up varies and often only in-hospital mortality is reported [1, 15, 34–40]. The 30-day and 6 month mortality rates in our cohort were 5.3 and 15.2% respectively, which are similar to outcomes in other units. Numerous studies have attempted to describe predictors of mortality in PLA; advanced age, leucocytosis, hyperbilirubinaemia, underlying malignancy, biliary aetiology, diabetes, abscess diameter ≥ 5 cm and underlying organism have all been suggested as risk factors [5, 22, 41–47]. In our study, only baseline ALP and the presence of loculation were predictive of mortality.

Along with antibiotic therapy, options for treatment of PLA include percutaneous aspiration, percutaneous drainage and open surgical drainage. There have been no trials that have compared these different treatment strategies. In our group, 60% of cases were managed with aspiration or drainage in addition to antibiotic therapy (76.3% of large (> 5 cm) abscesses vs. 25% of small (< 5 cm) abscesses). Seven (5.3%) of our PLAs required surgical drainage. It is unclear how percutaneous or surgical drainage affected outcomes. Antibiotic treatment was altered at least once in 79.5% of PLA patients (more than twice in 34.1%), some due to antibiotic resistance, with 34.1% of patients growing a resistant organisms, including 12.1% resistance to one of the most commonly used empiric antibiotic, co-amoxiclav, but others will have been altered empirically due to deterioration of the patient. This observation underlines the importance of early pathogen identification in order to optimise management.

## Conclusions

There is little quality evidence to guide optimal management of PLA. Using a well-characterised cohort of liver abscess patients, we found some correlations between organism, clinical features and outcomes of treatment. The heterogeneity of this cohort of liver abscesses, as with others in the literature, means that associations may have been missed and that those identified, such as higher CRP in ALAs and larger size in PLAs caused by streptococci, are too subtle to be of use to clinicians. These findings emphasise the need for a microbiological diagnosis, which is most effectively obtained by culture of aspirate fluid. We found 16 s rRNA gene PCR useful in the identification of bacteria in culture negative samples. Newer molecular technologies such high-throughput sequencing may, in the future, further improve the sensitivity of nucleic acid amplification tests and may also provide antibiotic susceptibility data and this is likely to lead to improved outcomes for individuals with these infections.

## Additional file

Additional file 1: 4 tables showing additional supporting statistical analysis of data described in main manuscript. (DOCX 30 kb)

## Data Availability

The data on which this study was based can be made available by the corresponding author to *bone fide* researchers subject to appropriate ethical approvals.

## Abbreviations

ALA: Amoebic liver abscess
ALP: Alkaline phosphatase
ALT: Alanine transferase
BSAC: British Society of Antimicrobial Chemotherapy
CAP: Cellulose Acetate Precipitin
CPA: Clinical Pathology Accreditation
EUCAST: European Committee on Antimicrobial Susceptibility Testing
IFAT: Immunofluorescent antibody test
PLA: Pyogenic liver abscess
rRNA: Ribosomal RNA
