# Supplementary material for: Clinical characteristics and treatment outcomes in a cohort of patients with pyogenic and amoebic liver abscess
Source: BMC Infect Dis. 2019 Jun 3;19:490. doi: 10.1186/s12879-019-4127-8 (PMC6547479; doi:10.1186/s12879-019-4127-8)
Supplement: Supplementary file 1 — 4 tables showing additional supporting statistical analysis of data described in main manuscript. (DOCX 30 kb) [file 12879_2019_4127_MOESM1_ESM.docx]

**SUPPLEMENTARY MATERIAL**

**Table S1: Position of abscesses within the liver**

|  | Pyogenic (n=118) | Amoebic (n=9) | All (n=127) |
| --- | --- | --- | --- |
| Left lobe† | 21 (17.8%) | 0 (0.0%) | 21 (16.5%) |
| Right lobe | 81 (68.6%) | 7 (77.8%) | 88 (69.3%) |
| Both | 11 (9.3%) | 2 (22.2%) | 13 (10.2%) |
| Other | 5 (4.2) | 0 (0.0%) | 5 (3.9%) |

†”Left” defined as being described as in segments 2, 3, 4a and 4b on either CT or ultrasound imaging, “Right” defined as being in segments 5, 6, 7, 8

**Table S2: Method of pathogen detection in pyogenic liver abscess**

| **Method** | **n (%)** |
| --- | --- |
| Blood culture | 26 (19.7%) |
| Pus culture | 34 (25.8%) |
| Pus 16S PCR | 9 (6.8%) |
| Pus 16S PCR + pus culture  Blood culture + pus culture | 4 (3.03%)  6 (4.5%) |
| Pus 16S PCR + pus culture + blood culture | 1 (0.8%) |
| Other | 1 (0.8%) |
| No positive culture | 51 (38.6%) |
| All via aspirate | 45 (34%) |
| All via blood culture | 33 (25%) |

**Table S3: Treatment outcome by type of organism**^‡^

|  | |  | None | Enterobacteriaceae including *E. coli* | *Klebsiella* | *S. milleri* | Other *Strep* spp. | Anaerobe | Other | Amoebic |  |
| --- | --- | --- | --- | --- | --- | --- | --- | --- | --- | --- | --- |
|  | |  | (n=51) | (n=35) | (n=21) | (n=19) | (n=7) | (n=16) | (n=13) | (n=9) | p-value† |
| Hospital stay | (days) | | 17 (9, 31) | 17 (11, 29) | 23 (14, 32) | 19 (13, 29) | 15 (9, 21) | 17.5 (12, 34) | 17 (12, 31) | 7 (6, 9) | p=0.04 |
| Antibiotics | (days) | | 41.5 (18.5, 53.5), n=40 | 51 (45, 63), n=25 | 56.5 (51, 80), n=18 | 60 (43.5, 75.5), n=16 | 44.5 (37.5, 52.5), n=4 | 48 (41, 64), n=13 | 48 (44, 61.5), n=8 | 22 (18.5, 27), n=8 | p=0.04 |
| Number of deaths (all-cause, ≤30 days) | | | 2 (3.9%) | 3 (8.6%) | 1 (4.8%) | 1 (5.3%) | 1 (14.3%) | 2 (12.5%) | 2 (14.3%) | 0 (0.0%) | p=0.61 |
| Number of deaths (all-cause, ≤6 months) | | | 7 (13.7%) | 8 (22.9%) | 4 (19.1%) | 1 (5.3%) | 1 (14.3%) | 3 (18.8%) | 5 (35.7%) | 0 (0.0%) | p=0.30 |
| Days to death (all-cause, ≤6 months) | | | 42 (18, 47), n=7 | 38 (15, 82), n=8 | 36 (28, 111), n=4 | 4, n=1 | 21, n=1 | 21 (17, 184), n=3 | 34 (21, 64), n=5 | n=0 |  |
|  | |  |  |  |  |  |  |  |  |  |  |
| All-cause mortality rate (≤6 months) per 1,000 patient days (95% CI) | | | 7.42 (3.71, 14.8) | 10.9 (5.87, 20.3) | 6.66 (2.50, 17.7) | 5.62 (1.41, 22.5) | 8.93 (1.26, 63.4) | 5.85 (1.89, 18.1) | 13.4 (5.56, 32.1) | 0 |  |
| Hazard ratio (95% CI) for death (all-cause, ≤6 months, age-adjusted) | | | 1.00 (reference) | 1.20 (0.54, 2.69) | 0.93 (0.36, 2.39) | 0.88 (0.20, 3.75) | 1.29 (0.59, 2.79) | 0.87 (0.29, 2.62) | 1.28 (0.59, 2.79) | - | p=0.87 |

† For continuous measures, F statistic comparing Somer’s D parameters for association of measure with organism type, adjusted for clustering within patients; for categorical variables, Fisher’s exact test; for hazard ratios, Wald-type test (6 d.o.f)

**Table S4: Factors associated with death (all-cause, within 6 months)**

|  |  | Age-adjusted hazard ratio (95% CI) | Hazard ratio (95% CI) adjusted for age and included variables† |
| --- | --- | --- | --- |
| Sex | Male | 0.99 (0.42, 2.33) |  |
| Number of microorganisms | 0 | 1.00 (ref) |  |
|  | 1 | 1.10 (0.42, 2.86) |  |
|  | 2 | 0.44 (0.05, 3.67) |  |
|  | 3 | 0.66 (0.13, 3.21) |  |
|  | 4 | 5.46 (0.63, 47.7) |  |
| Number of abscesses | 1 | 1.00 (ref) |  |
|  | 2 | 0.62 (0.15, 2.55) |  |
|  | 3+ | 1.27 (0.49, 3.32) |  |
| Maximum diameter | per cm | 0.91 (0.76, 1.08) |  |
| Loculated | Yes *vs.* No | 1.93 (0.79, 4.71) | 2.51 (1.00, 6.32) |
| Position | Right | 0.43 (0.11, 1.61) |  |
|  | Left | 1.00 (ref) |  |
| Baseline anaemia | HB <110g/L | 1.98 (0.83, 4.72) |  |
| Baseline WBC | per log_10_ increase | 2.06 (0.19, 22.5) |  |
| Baseline neutrophils | per log_10_ increase | 1.53 (0.18, 13.0) |  |
| Baseline CRP | per log_10_ increase | 0.67 (0.27, 1.64) |  |
| Baseline ALT | per log_10_ increase | 0.83 (0.35, 1.97) |  |
| Baseline ALP | per log_10_ increase | 3.42 (0.84, 13.8) | 4.78 (1.19, 19.2) |
| Baseline BILI | per log_10_ increase | 1.49 (0.44, 5.07) |  |
| Diabetes | Yes *vs.* No | 1.48 (0.59, 3.72) |  |
| Immunosuppression | Yes *vs.* No | 2.26 (0.73, 7.05) |  |
| Other pathology* | None | 1.00 (ref) |  |
|  | Biliary malignancy | 1.89 (0.62, 5.77) |  |
|  | Other biliary pathology | 1.37 (0.29, 6.43) |  |
|  | Non-malignant GI | 1.02 (0.30, 3.44) |  |
|  | Other cancer | 3.33 (0.23, 48.9) |  |
| Antibiotic resistance^‡^ | Yes *vs.* No | 1.13 (0.49, 2.60) |  |

† Adjusted for age as a continuous variable, and two of the four variables carried forward (loculated and baseline ALP) which were associated (p≤0.05) with the outcome after mutual adjustment (anaemia and immunosuppression were not associated with the outcome when adjusted for any of the other variables)

* Biliary Malignancies (cholangiocarcinoma); Other biliary pathology (gallstones, cholangitis/cholecystitis); Non malignant GI pathology (diverticulitis, fistula, portal vein thrombosis); Other cancer (metastatic melanoma, colorectal cancer, pancreatic cancer)

^‡^ Coded ‘yes’ if any isolated microorganism resistance to one or more antibiotics
